# Toward the prevention of clinically manifest Parkinson’s disease

**DOI:** 10.1007/s00702-026-03240-x

**Published:** 2026-08-04

**Authors:** Günter Höglinger, Franziska Hopfner

**Affiliations:** 1 Department of Neurology, LMU University Hospital, Ludwig-Maximilians-Universität (LMU) München, Marchioninistr. 15, 81377 Munich, Germany; 2 German Center for Neurodegenerative Diseases (DZNE), Munich, Germany; 3 Munich Cluster for Systems Neurology (SyNergy), Munich, Germany; 4 Aligning Science Across Parkinson’s (ASAP) Collaborative Research Network, Chevy Chase, MD USA

**Keywords:** Parkinson’s disease, Biomarkers, Precision neurology, α-synuclein, Artificial intelligence, Prevention, Digital twins, Public health

## Abstract

Parkinson’s disease (PD) is the fastest growing neurological disorder worldwide and is projected to affect unprecedented numbers of individuals by 2050. At the same time, PD research is undergoing a profound conceptual transformation. Advances in molecular neuropathology, genetics, biomarker science, artificial intelligence, multimodal imaging, digital medicine, and precision therapeutics are reshaping PD from a clinically defined syndrome into a biologically measurable and potentially preventable process. This Perspective is not a report of new empirical findings and should not be read as an evidence-based forecast of what will most likely occur by 2050. Rather, it deliberately formulates a normative strategic vision: clinically manifest PD should become a preventable public-health burden. In this context, “eradication” does not imply elimination of all α-synuclein pathology, genetic susceptibility, or neurodegenerative biology. It refers to the long-term objective of preventing or substantially eliminating the transition to disabling, clinically manifest PD before irreversible symptomatic neurodegeneration occurs. We outline the prerequisites for such a future, including validated biological staging, risk stratification, longitudinal biomarker trajectories, trial enrichment, preventive endpoints, regulatory qualification, scalable implementation, and equitable global access. The analogy to poliomyelitis is used as a model of strategic goal-setting and international coordination, not as a biological equivalence between an infectious disease and a heterogeneous neurodegenerative disorder. Several enabling technologies already exist in early or research form, including α-synuclein seed amplification assays, genetic and prodromal risk models, digital monitoring, multimodal imaging, and mechanism-based therapeutic development. Other components remain aspirational and will require major scientific, regulatory, ethical, and societal advances. The central purpose of this article is therefore not to predict the future from the current trajectory, but to define a desired destination around which the field can organize scientific innovation.

## Introduction: why we need a new vision

Parkinson’s disease (PD) is currently the fastest growing neurological disorder worldwide and represents a major and increasing public health challenge (Dorsey and Bloem 2018). Current projections suggest that the number of affected individuals will continue to rise substantially until at least 2050 (Su et al. 2025).

More than 200 years after James Parkinson first described the “Shaking Palsy,” the diagnosis of PD still largely depends on the emergence of clinically manifest disease, defined here as the motor and/or non-motor syndrome that meets established diagnostic criteria and generates symptomatic burden requiring medical care. However, neurodegeneration begins many years before overt motor symptoms appear (Postuma et al. 2016; Poewe et al. 2017).

The classical trajectory of PD includes prolonged prodromal stages characterized by non-motor manifestations such as REM sleep behavior disorder, constipation, hyposmia, autonomic dysfunction, anxiety, depression, fatigue, and subtle cognitive impairment (Poewe et al. 2017). By the time classical motor symptoms emerge, substantial nigrostriatal degeneration has already occurred.

The last decades of PD research have achieved major advances, including the development and refinement of dopamine replacement therapies; the implementation of deep-brain stimulation and infusion-based therapies; a broader understanding of genetic and environmental contributors; the recognition of prion-like propagation of α-synuclein pathology; and the characterization of progression from prodromal manifestations to motor and cognitive dysfunction (Bloem et al. 2021; Ben-Shlomo et al. 2024; Foltynie et al. 2024; Morris et al. 2024).

The field is now undergoing a profound transformation, driven by molecular neuropathology, biomarker discovery, proteomics and (epi-)genomics, multimodal imaging, digital medicine, artificial intelligence, and precision therapeutics. These developments collectively raise a provocative question: should the field organize itself around preventing clinically manifest PD rather than only improving treatment after diagnosis?

This article intentionally adopts an ambitious but explicitly normative perspective. It does not forecast that clinically manifest PD will inevitably be eradicated by 2050. Instead, it defines a strategic endpoint that can guide the selection of scientific, clinical, regulatory, and public-health priorities. The distinction matters: a forecast extrapolates from the current path, whereas a vision statement defines the destination and motivates the search for a path toward it.

The analogy to the eradication of poliomyelitis refers to strategic goal-setting, surveillance, international coordination, and sustained investment rather than to disease mechanism. In this sense, “eradication” is used pragmatically to mean the prevention or major population-level elimination of clinically manifest PD as a disabling condition.

## From syndromic neurology to molecular disease definitions

Historically, neurodegenerative diseases have largely been classified according to clinical syndromes. Parkinson’s disease, multiple system atrophy, dementia with Lewy bodies, progressive supranuclear palsy, corticobasal degeneration, and frontotemporal dementia were primarily differentiated on the basis of symptomatology and neuropathology.

Modern molecular neuropathology increasingly demonstrates that neurodegenerative diseases are proteinopathies characterized by distinct conformational strains with anatomical, cellular, and morphological aggregation patterns (Rodriguez et al. 2015; Schweighauser et al. 2020; Zhang et al. 2020; Shi et al. 2021; Scheres et al. 2023). Synucleinopathies are associated with α-synuclein aggregation, tauopathies with tau aggregation, and amyloid-β, TDP-43, and FUS pathology characterize other major disorders.

This represents a paradigm shift from symptom-based disease definitions toward biologically defined molecular entities (Tabrizi et al. 2022; Jack et al. 2024; Höglinger et al. 2024; Simuni et al. 2024; Palleis et al. 2026).

However, biological classification will not replace clinical neurology; it must be integrated with it. Mixed pathologies are common in ageing brains, clinical manifestations can vary substantially even among individuals with similar genetic or pathological backgrounds, and co-pathologies involving tau, amyloid-β, vascular injury, or TDP-43 may modify phenotype and progression. A 2050 framework must therefore combine biological definitions with clinical phenotyping, functional burden, patient-reported outcomes, and longitudinal trajectories.

By 2050, diagnosis of PD may rely increasingly on characterization of α-synuclein aggregation propensity and strain specification, genomic and epigenomic signatures, proteomic and metabolomic fingerprints, immune phenotyping, microbiome analyses, and multimodal imaging. Neurological taxonomy may ultimately resemble modern molecular oncology more closely than classical descriptive neurology, but it must remain anchored to clinical meaning and patient benefit.

## Earlier than early: preclinical detection

One of the most important conceptual advances in PD research is the recognition that disease biology begins years to decades before motor diagnosis (Postuma et al. 2016; Poewe et al. 2017). Existing MDS prodromal PD criteria and newer biological classification frameworks already provide research structures for this preclinical space (Berg et al. 2015; Heinzel et al. 2019; Höglinger et al. 2024; Simuni et al. 2024).

Future disease models may distinguish genetic susceptibility, environmental and exposomal challenge, biomarker-positive preclinical PD, early biological PD, prodromal non-motor PD, and clinically manifest PD. This expanded disease timeline creates unprecedented opportunities for preventive intervention, but also requires careful validation before individual-level risk predictions are returned in routine care.

## Risk profiling across the lifespan

The number of known PD-associated genetic loci continues to increase, and polygenic risk scores may eventually contribute to individualized estimation of lifetime neurodegenerative risk. The revised framing is deliberately cautious: broad genomic risk profiling, whether at birth or later in life, should be considered only when clinical utility, counseling, privacy protection, and equitable access are established. Potential applications include personalized monitoring, environmental risk reduction, and selective enrolment into prevention studies.

Genetics alone cannot explain PD pathogenesis in most individuals (Hopfner et al. 2020). Environmental influences likely interact with genetic susceptibility through epigenetic regulation. Future preventive medicine may therefore include periodic epigenomic or immune-metabolic reassessment to identify toxin-associated molecular signatures, inflammatory activation, accelerated biological aging, or evolving risk trajectories.

The exposome encompasses the cumulative environmental exposures experienced throughout life. Increasing evidence suggests that pesticides, air pollution, heavy metals, industrial chemicals, and microbiome alterations contribute to PD risk (Dorsey and Bloem 2018). By 2050, environmental surveillance, geospatial exposure mapping, dietary tracking, and microbiome profiling may support public-health prevention strategies (Ibanez et al. 2025; Okubadejo et al. 2026). These approaches should be framed as population-risk reduction tools, not deterministic predictions for individuals.

## Biomarkers and biological staging

The development of biomarker-driven disease models in Alzheimer’s disease provides a blueprint for the future of Parkinson medicine (Hansson 2021; Jack et al. 2024). Fluid biomarkers, seed amplification assays, PET imaging, exosomal profiling, proteomics, and digital measures are rapidly transforming PD research. The objective of future medicine will not simply be “early clinical diagnosis,” which often remains late from a biological perspective, but biological interception before irreversible neurodegeneration occurs.

For evidentiary clarity, candidate biomarkers should be separated into three categories. First, clinically emerging or research-validated tools include α-synuclein seed amplification assays, selected genetic markers, prodromal risk criteria, dopamine transporter imaging, and established neurodegeneration markers such as neurofilament light chain. Second, translational research tools include blood-based proteomic, metabolomic, inflammatory, mitochondrial, lysosomal, and extracellular vesicle signatures, as well as multimodal imaging markers. Third, long-horizon concepts include fully individualized multi-omic trajectories, population-level biological screening, and integration of biomarker dynamics into digital twins.

The limitations of current biomarker science must be stated explicitly. Assay reproducibility, cross-platform harmonization, pre-analytical variability, longitudinal stability, tissue and fluid comparability, biological heterogeneity, and prediction of conversion from prodromal states remain major challenges. False positives, false negatives, and uncertain prognostic meaning are particularly relevant if biomarkers are used outside enriched research cohorts. A prevention strategy can therefore be justified only if analytical validity, clinical validity, clinical utility, and ethical implementation are established stepwise.

While biological monitoring and screening may become integrated into routine preventive healthcare programs, detection of subtle neurological dysfunction suggestive of incipient clinically manifest PD may increasingly be supported by digital systems monitoring speech, language, mobility, sleep, activity, and behavioral responsiveness through smartphones, smart watches, home environments, and vehicles (Falkenburger et al. 2026). Such systems should augment, not replace, clinical judgment.

## Digital twins and computational neurology

In neurology, an individualized digital twin should be defined operationally as a continuously updated computational model that integrates longitudinal biological, clinical, behavioral, imaging, environmental, and treatment data to estimate risk, simulate plausible trajectories, and support shared decisions. Without this operational definition, the term risks becoming a buzzword rather than a testable proposal.

Required inputs would include wearable sensor data, gait and speech analytics, sleep recordings, eye movement tracking, multimodal imaging, genomic and proteomic data, biomarker trajectories, medication exposure, adverse events, comorbidities, and environmental exposures. Outputs would include estimates of conversion risk, progression velocity, treatment response, adverse-event risk, and individualized monitoring intervals.

Validation should be prospective and clinically meaningful. Acceptable standards would include calibration across diverse populations, comparison with clinician-based prediction, pre-specified endpoints, transparent error estimates, bias audits, and demonstration that digital-twin-supported decisions improve outcomes or reduce burden. Neurology may thereby evolve from episodic clinic-based care toward continuous predictive medicine, but only if these systems remain accountable, interpretable, secure, and embedded in physician–patient decision-making.

## Precision therapy and mechanism-based medicine

Current PD treatment still largely follows a symptomatic strategy. Increasing evidence suggests that PD encompasses multiple biological subtypes characterized by distinct mechanisms, including mitochondrial dysfunction, endo-lysosomal or autophagic impairment, LRRK2 hyperactivity, α-synuclein aggregation, gut microbiome dysregulation, neuroinflammation, iron metabolism disturbances, and co-pathologies involving tau or amyloid-β (Stocchi et al. 2024).

A clearer therapeutic framework distinguishes four levels: disease-modifying therapy in biologically defined PD, preventive therapy in high-risk or biomarker-positive individuals, adaptive monitoring to escalate or de-escalate therapy, and adjunct symptomatic or device-assisted care for individuals who develop clinical manifestations.

## Disease-nodifying and preventive therapy

Mechanism-based therapies may target α-synuclein aggregation, lysosomal dysfunction, mitochondrial biology, neuroinflammation, iron handling, synaptic resilience, or genetically defined pathways. Preventive therapy would require a higher evidentiary threshold than symptomatic treatment because it may be offered before disability occurs. Trials should therefore enrich participants by biological risk, use sensitive biomarker and digital endpoints, and demonstrate a favorable long-term benefit-risk ratio.

Gene editing or transfection technologies such as CRISPR/Cas or persistent viral expression of shRNAs or proteins may eventually permit correction of pathogenic mutations, modulation of risk genes, enhancement of neuroprotective pathways, or suppression of pathological protein expression. These approaches remain experimental and should be presented as long-horizon possibilities rather than near-term clinical promises.

## Biomarker-guided adaptive therapy

Future treatment strategies may rely on continuous biomarker monitoring. Therapy selection, escalation, de-escalation, combination, or switching could be guided by α-synuclein biomarker dynamics, markers of disease activity, inflammatory signatures, imaging progression, adverse-event risk, and digital phenotyping. This approach would parallel adaptive precision oncology but must be adapted to the slower time course, uncertain surrogate endpoints, and long preclinical phase of neurodegeneration.

## Future symptomatic and device-assisted therapies

Even if disease-modifying therapies emerge, symptomatic treatment will continue evolving. Likely developments include adaptive closed-loop deep brain stimulation, directional deep brain stimulation systems, minimally invasive neuromodulation, advanced infusion therapies, MR-guided focused ultrasound, and AI-guided neurostimulation algorithms. Device-assisted therapies will likely become increasingly individualized based on neurophysiological and imaging data.

## Prevention and the vision of eradication

The concept of eradicating clinically manifest PD may initially appear unrealistic. However, history demonstrates that ambitious public-health objectives can catalyse transformative scientific progress. The near-eradication of poliomyelitis was achieved through surveillance, vaccination, rapid outbreak response, international cooperation, and sustained public investment.

For PD, an analogous framework would not be based on interrupting transmission, but on identifying biological risk early, reducing avoidable environmental exposures, intervening before irreversible neurodegeneration, and ensuring long-term monitoring. The analogy is therefore strategic and organizational, not mechanistic.

Some potential interventions are close to current translational science, including biomarker-guided trial enrichment, lifestyle optimization, targeted reduction of environmental neurotoxin exposure, and mechanism-based therapies for genetically or biologically defined subgroups. Other concepts, such as preventive immunization, microbiome engineering, targeted regional neuroprotective interventions, or wastewater monitoring for pathological α-synuclein species, should be framed explicitly as exploratory frontiers or thought experiments rather than near-term policy proposals.

The central point is not that all of these approaches will succeed. The central point is that PD should increasingly be treated as a preventable public-health challenge rather than only as an individual symptomatic disorder encountered after motor onset.

### Ethical, legal, and societal challenges

The future described here introduces ethical, legal, and societal challenges that are central rather than peripheral. Preventive neurology would create large groups of individuals who may be biologically or genetically at risk but not ill. Communicating such risk before actionable interventions exist could cause anxiety, stigma, altered self-identity, and unnecessary medicalization.

Risk disclosure will require robust counseling, shared decision-making, and careful thresholds for returning results. Genetic determinism must be avoided: polygenic risk, α-synuclein biomarker positivity, or prodromal markers should be presented probabilistically, with explicit discussion of uncertainty and modifiable risk.

Lifelong monitoring raises questions about consent, data ownership, privacy, cybersecurity, secondary use of biological and digital data, and the right not to know. Strong safeguards will be required to prevent discrimination by employers, insurers, or other institutions based on genetic, biomarker, or algorithmic risk.

Algorithmic systems may reproduce or amplify bias if trained mainly in high-resource, European-ancestry, or highly selected cohorts. Therefore, any digital twin, risk score, or biomarker algorithm must be validated across sex, age, ancestry, geography, socioeconomic status, comorbidity, and health-system contexts.

Equity must be built into prevention from the beginning. Population-level screening, multimodal biomarkers, and precision therapeutics could widen global disparities if they are expensive, centralized, or available only in wealthy countries. Scalable assays, tiered screening strategies, open standards, global capacity building, and affordability should therefore be treated as scientific requirements rather than implementation afterthoughts.

## A call to action

**Fig. 1:**
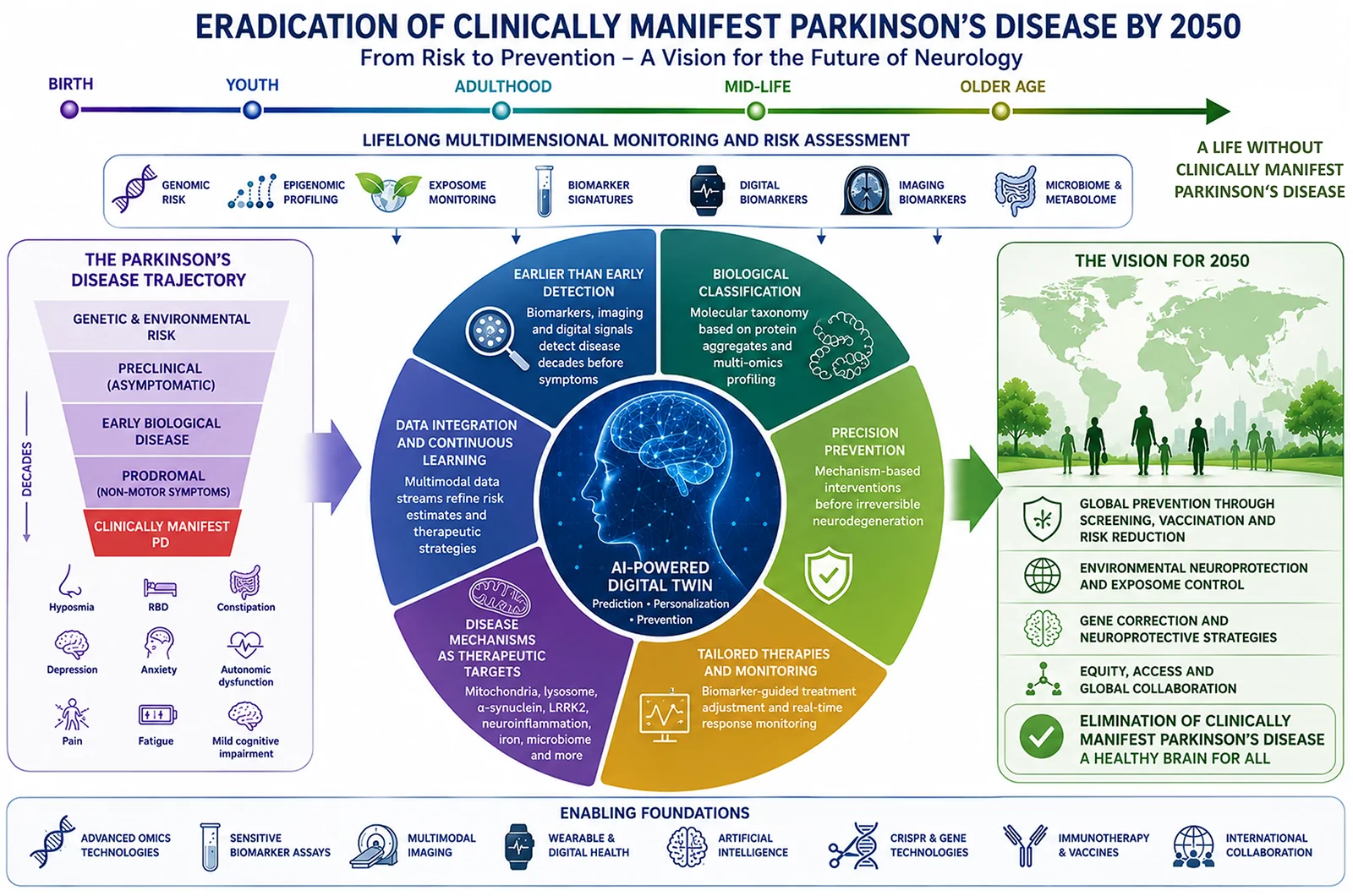
Conceptual framework for preventing clinically manifest Parkinson’s disease as a public-health burden. The revised figure separates the individual life-course axis from the strategic 2050 objective, addressing the reviewer’s concern that “2050” could otherwise be misconstrued as the endpoint of an individual life-course trajectory (generated with ChatGPT based on the author’s intellectual input)

If clinically manifest PD is to become preventable, incremental progress will not suffice. The neurological community should prioritize global longitudinal biomarker cohorts, standardized biological staging systems, prevention-focused clinical trials, regulatory qualification of biomarkers and digital endpoints, integration of trustworthy artificial intelligence into routine neurology, WHO-level international neurodegeneration initiatives, environmental neuroprotection policies, equitable global access to biomarkers and therapies, and interdisciplinary collaboration across neuroscience, engineering, public health, ethics, economics, and computational biology. The greatest danger for the field is not excessive ambition, but insufficient ambition (see Fig. 1).

## Conclusion

**Fig. 2:**
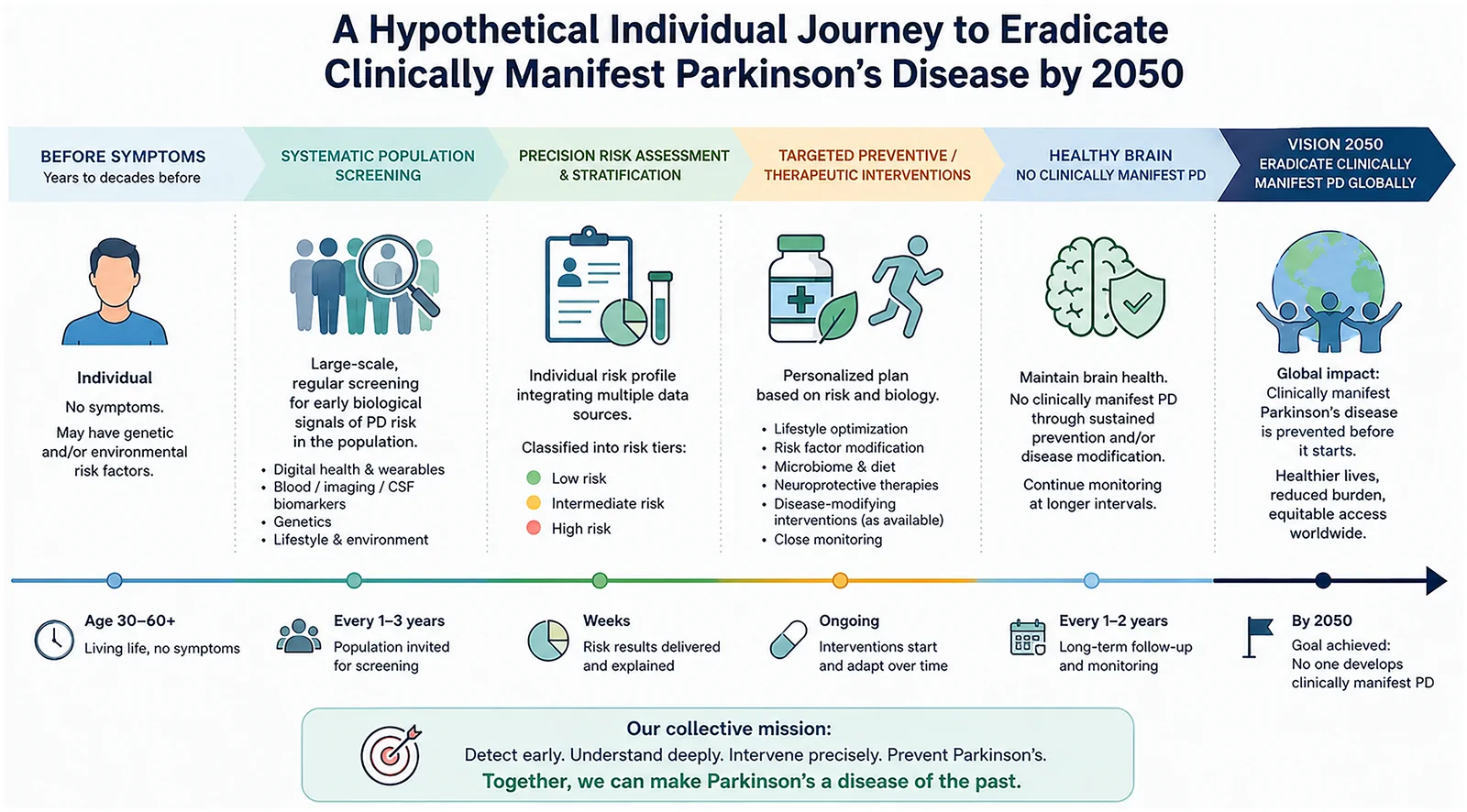
Hypothetical individual screening, coaching, treating, and monitoring programme to prevent clinical manifestation of Parkinson’s disease. The figure illustrates a desired prevention pathway and should be interpreted as a strategic vision rather than a prediction that every element will be implemented (generated with ChatGPT based on the author’s intellectual input)

The next 25 years may fundamentally redefine PD. It is plausible that PD will no longer be diagnosed primarily through tremor, rigidity, and bradykinesia, but through molecular fingerprints, biomarker trajectories, digital phenotypes, and individualized risk signatures detectable before symptom onset (Fig. 2). Therapy may become more preventive than reactive, more biological than syndromic, and more personalized than empirical.

Whether clinically manifest PD can ultimately be prevented as a major public-health burden by 2050 cannot be predicted today. The purpose of this article is therefore not to provide a weather forecast for Parkinson medicine. It is to define a destination: a future in which disabling PD is avoided whenever possible through early detection, biological understanding, precise intervention, ethical governance, and global access. In that sense, eradication of clinically manifest PD should be understood not as an epidemiological claim that all disease biology will disappear, but as a strategic commitment to make symptomatic neurodegeneration increasingly preventable.

## Data Availability

No datasets were generated or analysed during the current study.
